# Increased risk of somatic diseases following anorexia nervosa in a controlled nationwide cohort study

**DOI:** 10.1002/eat.23718

**Published:** 2022-04-22

**Authors:** Hans‐Christoph Steinhausen, Martin Dalgaard Villumsen, Kirsten Hørder, Laura Al‐Dakhiel Winkler, Niels Bilenberg, René Klinkby Støving

**Affiliations:** ^1^ Department of Child and Adolescent Mental Health Odense, Mental Health Services in the Region of Southern Denmark University of Southern Denmark Odense Denmark; ^2^ Child and Adolescent Mental Health Centre, Capital Region Psychiatry Copenhagen Denmark; ^3^ Department of Child and Adolescent Psychiatry Psychiatric University Hospital of Zurich Zurich Switzerland; ^4^ Clinical Psychology and Epidemiology, Institute of Psychology University of Basel Basel Switzerland; ^5^ Department of Epidemiology, Biostatistics, and Biodemography, Institute of Public Health University of Southern Denmark Odense Denmark; ^6^ Center for Eating Disorders Odense University Hospital Odense Denmark; ^7^ Psychiatric Services in the Region of Southern Denmark Odense Denmark; ^8^ Endocrine Research Unit Odense University Hospital Odense Denmark

**Keywords:** anorexia nervosa, cohort study, comorbidity, somatic disease

## Abstract

**Objective:**

To assess the risk of somatic diseases in connection with anorexia nervosa (AN).

**Method:**

This matched cohort study was based on Danish registries of all patients born 1961–2008 with a first‐time diagnosis of AN in 1994–2018 at age 8–32 and matched controls without an eating disorder. For 13 somatic disease categories, time from inclusion date to time of first somatic diagnosis, accounting for censoring, was studied by use of time‐stratified Cox models.

**Results:**

A total of 9985 AN patients born 1961–2008 and 49,351 controls were followed for a median (interquartile range) of 9.0 (4.4–15.7) years. During the first 2 years after entry there was a 60% higher hazard for any somatic disease among patients with AN than among controls, while the ratio from three to 11 years was reduced to 1.18. Regardless of age at diagnosis, the hazard among patients and controls were no different at approximately a decade after diagnosis of AN and the cumulative risk for patients for 12 of 13 disease categories was always higher or no less that for controls. For all disease categories, the hazard ratio (HR) was higher when close to entry. For most disease categories, age at diagnosis of AN did not modify the effect.

**Discussion:**

While around 90% of all individuals had any somatic disease at the end of follow‐up, the cumulative incidence over time was higher for patients with AN than for controls. Large HRs were seen in the early years after diagnosis during which patients require extensive medical interventions.

**Public Significance:**

Based on Danish registries, a large sample of almost 10,000 patients with AN born 1961–2008 and almost 50,000 matched controls were followed for a median of 9 years. While around 90% of all individuals had any somatic disease at the end of follow‐up, the cumulative incidence over time was higher for patients with AN than for controls.

## 1 INTRODUCTION

In the Global Burden of Disease study published from 2013, eating disorders (ED) were ranked 12th among 305 diseases in women with the highest disability adjusted life years (Erskine et al., 2016; GBD 2013 DALYs and HALE Collaborators, 2015). The inherent symptoms of anorexia nervosa (AN) can ultimately lead to various severe somatic complications (Forney et al., 2016; Katzman, 2005; Mitchell & Crow, 2006) including high risks of cardiovascular complications (Sachs et al., 2016), gastrointestinal complications (Norris et al., 2016), bone diseases (Frolich et al., 2017; Misra et al., 2016), heightened fracture risk (Frolich et al., 2020; Vestergaard et al., 2002), renal complications (Stheneur et al., 2014), obstetric and gynecologic problems (Andersen & Ryan, 2009; Kimmel et al., 2016), and orofacial manifestations (Romanos et al., 2012). It has also been proposed that AN and autoimmune disease might have an underlying common etiology (Raevuori et al., 2014) supported by the skewed gender distribution for both diseases (Acres et al., 2012; Fetissov et al., 2002; Quintero et al., 2012). Studies focusing on diabetes mellitus (DM) found an increased prevalence of disordered eating and some ED but not of AN in DM‐type 1 (Hanlan et al., 2013; Mannucci et al., 2005). A recent study based on Danish registry data found that medical condition comorbidity is increased among those with ED (Momen et al., 2021).

The outcome and prognosis for AN per se is poor, with only less than half of patients attaining full recovery (Steinhausen, 2002, 2009; Steinhausen et al., 2003). In a sizable number of outcome studies, onset during adolescence was associated with a more benign outcome than onset in adulthood (Steinhausen et al., 2003), but there is renewed evidence from a nationally representative US study that onset in childhood (defined as <15 years) is associated with more severe AN, greater life difficulties, and greater lifetime psychiatric comorbidity than in later ages (Grilo & Udo, 2021). So far, the course and outcome of patients in terms of a wider spectrum of comorbid somatic diagnoses (Erdur et al., 2012; Wentz et al., 2012) in contrast to psychiatric disorders (Steinhausen, 2009; Steinhausen et al., 2021) is not well‐examined. Somatic comorbidity may sometimes require transfer to intensive care (Stheneur et al., 2017) and is associated with poor outcome and mortality (Erdur et al., 2012). Moreover, there is evidence that a wide range of somatic illnesses are more prevalent in patients with other severe mental illnesses including schizophrenia, bipolar disorders, and major depression, compared to the general population (De Hert et al., 2011).

The present study is the second based on register data from a complete nationwide Danish cohort of patients with AN focusing on course and outcome. In the preceding paper we focused on mental comorbidities (Steinhausen et al., 2021). The aim of the present paper was to study the time to any first somatic disease following AN compared to matched controls considering that age at onset (or first diagnosis) of AN could be an effect modifier of somatic comorbidity as it has been shown to be a moderator of clinical outcome in uncontrolled studies (Steinhausen, 2002, 2009).

## 2 METHOD

### 2.1 Study samples

The study was based on the nationwide cohort of all individuals born from 1961 to 2008, who at the age of 8–32 were first‐time diagnosed with ED between the years of 1994 and 2018. ED were defined by the International Classification of Diseases and Related Health Problems, 10th edition (ICD‐10) (WHO, 1992), code F50 in the National Patient Register (NPR) which is not quite the same as what was included in DSM‐5. A flowchart of the sample populations has been published with the preceding companion paper (Steinhausen et al., 2021). The final sample amounted to 9985 patients with the first diagnosis of AN (F 50.0 AN and F 50.1 atypical AN) and to 49,351 controls. According to sex assigned at birth, patients with AN were predominantly female (93.5%). The dataset did not contain information on race/ethnicity and socioeconomic status.

Patients with AN were identified in the registers as individuals with at least one AN diagnosis at the first day of registration with a nonemergency incident ED diagnosis, regardless of whether the patient simultaneously had a bulimia nervosa diagnosis as well. As was argued in our preceding paper (Steinhausen et al., 2021), the definition of the age range was guided by the idea of uncertainty in regards to the validity of AN diagnosis before 8 years and from the early 30s. The sample was divided into three subgroups by age in years at first diagnosis of AN, covering 8–13, 14–17, and 18–32 at the time of entry. The three subgroups were defined according to common developmental distinctions between childhood including preadolescence (age 8–13), adolescence (age 14–17), and adulthood (age 18 and older).

The sample has already been defined in the preceding companion paper on mental comorbidity. To avoid prevalent cases with AN from being classified as incident cases, all cases with mental disorders (including AN) and contact with psychiatric services before 1994 were excluded by study design. This procedure, which was used separately for each disorder, implies an exclusion of all patients and controls with the ICD‐8 classification. In Denmark, the ICD‐8 classification was used from 1969 to 1993 and the ICD‐10 was used from 1994. The cohort of AN patients was matched in a 1:5 ratio on sex and age at the time of first AN diagnosis to alive controls identified in the Danish Civil Registration System (CRS). Controls had not had any ED diagnosis at the end of follow‐up. All participants resided in Denmark when they were included in the study.

### 2.2 Procedure

The study was based on the comprehensive Danish system of registries with mandatory recording of each patient visit to the public health since 1968 (WHO, 1992). In the present study, based on the personal identification number (CPR) assigned to all Danish citizens and residents, information was collected from the CRS on date of birth, sex, postcode of residence at the time of the first diagnosis in the NPR. Furthermore, data on first‐time AN diagnosis, date, and type of admission to psychiatric facilities, were collected from the Danish Psychiatric Central Research Register (Mors et al., 2011). Data were provided by Statistics Denmark in an anonymized fashion and approval of the study was given by the Danish Data Protection Agency (file no. 15/280490). According to Danish law, ethical approval is not required for registry‐based studies. Access to the data requires application to the Danish authorities.

### 2.3 Outcomes

Thirteen different time‐to‐event outcomes were studied with AN onset at time zero and first somatic diagnoses as events. The entire somatic morbidity as represented by the 11 broad ICD‐10 categories of diseases (WHO, 1992) was considered in the analyses. In addition, the category of autoimmune diseases following the classification by Raevuori et al. (2014) and the collection of all categories (any somatic disease) were also considered. Given the fact that the outcomes were 13 broad somatic disease categories representing the entire range of specific diseases with a large variety of courses, no attempt was made to exclude cases with an incidence of any somatic disease prior to the first diagnosis of AN. Such an exclusion would have resulted in the loss of a major part of both samples and was not mandatory for the aim of studying time from inclusion date to time of first somatic diagnosis following first diagnosis of AN. All codes based on the ICD‐10 and the ICD‐8 classification are shown in Table S1. All diagnoses were made by the attending physicians.

### 2.4 Statistical analyses

Time to 12 categories of somatic disorders as well as all combined was investigated by use of time‐to event analyses that accounted for censoring. AN patients were compared to matched controls using Cox proportional hazards models with cluster robust standard errors. Date of entry for the AN patients was defined by the date of first diagnosis of AN; for the matched controls it was that of the corresponding patient with AN. Time until first diagnosis was analyzed for each of the 13 defined categories. Separate effects were estimated on periods for which time invariance was assessed with Aalen's linear hazard models (Lee & Weissfeld, 1998). The Cox models were adjusted for sex, period of entry (1994–1999, 2000–2004, 2005–2009, 2010–2014, 2015–2018), and family income (in tertiles) at entry based on the following considerations. There is an extremely uneven sex distribution in AN and the two sexes also show different attendance rates in the health system. Furthermore, period of entry was controlled for due to varying incidence rates of AN in Denmark (Steinhausen & Jensen, 2015), and family income was regarded as an indicator of socioeconomic status, which is an important covariate of disease. The assumptions of proportional hazards were verified via Schoenfeld residuals tests. Tests for interaction between entry age group and AN (patients vs. controls) for each mental disorder were conducted. The method proposed by Holm (1979) was used to correct for multiple testing. The risk of each of the 12 somatic diseases, and of any of them, over time in patients and controls were compared using cumulative incidences, with death as the only competing risk, for each age subgroup as well as the combined sample. To guarantee anonymity of individuals, cumulative incidences are graphed after applying a multiplicative distortion of a Gaussian distributed random error with mean 1 and standard deviation .05. All analyses were performed with Stata 16.1 (StataCorp) on servers at Statistics Denmark.

## 3 RESULTS

The baseline characteristics of each group are shown in Table 1. Patients with AN were predominantly female (93.5%). The largest age group (45%) was 18–32 years old, while 38% were 14–17 and 17% were 8–13 years old. Median (interquartile range) follow‐up time in years for the two samples was 9.0 (4.4–15.7). At the time of entry, the AN sample consisted of 1349 (13.5%) inpatients, 8595 (86.1%) outpatients and a negligible number of 41 patients in a day clinic (.4%). As controls were matched on age defined in years, the true age at entry for controls exceeded the age range 8–32 (range: 7.6–33.9). Among patients and controls, .7%, respectively, .2% were lost because of death.

**TABLE 1:** Sample characteristics

	Patients	Controls
Total	9985	49,351
Sex, no. (%)		
Female	9331 (93.5)	46,103 (93.4)
Male	654 (6.5)	3248 (6.6)
Age in years at inclusion, median (IQR)	17.3 (14.7–21.3)	17.3 (14.7–21.3)
Age groups in years		
8–13	1724 (17.3)	8519 (17.3)
14–17	3809 (38.1)	18,848 (38.2)
18–32	4452 (44.6)	21,984 (44.5)
Family income group (tertiles)		
Lower tertile	3477 (34.8)	16,301 (33.0)
Middle tertile	3011 (30.2)	16,768 (34.0)
Higher tertile	3497 (35.0)	16,282 (33.0)
Period of inclusion, no (%)		
1994–1999	1441 (14.4)	7075 (14.3)
2000–2004	1595 (16.0)	7835 (15.9)
2005–2009	1971 (19.7)	9726 (19.7)
2010–2014	2725 (27.3)	13,537 (27.4)
2015–2018	2253 (22.6)	11,178 (22.6)

Abbreviation: IQR, interquartile range.

**FIGURE 1:**
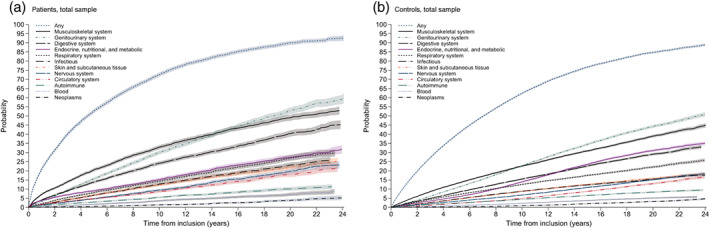
Cumulative incidences with 95% confidence intervals for somatic disease categories of patients with anorexia nervosa (AN) and controls

For documentation, regardless of censored individuals, the frequencies of somatic diseases for the entire observation period for the total samples and the various age groups are listed in Table S2. Cumulative incidence curves for each of the 13 diagnostic groups of somatic diseases for patients and controls are shown in Figure 1 for the total sample and in Figure S1 for the three age groups 8–13, 14–17, and 18–32. The curves indicate, regardless of diagnostic category and age group, that one out of two will get any somatic disease during the first 5–6 years after entry, while after two decades it is 9 out of 10. Across time, cumulative incidence for both samples and for all age groups were highest for musculoskeletal diseases, genitourinary diseases, and digestive diseases. Generally, the curves indicate slightly higher cumulative incidence over time for patients than for controls. For the total sample, there is a slightly higher cumulative incidence for AN patients at about 16 years after entry for endocrine, nutritional, and metabolic diseases. For the other categories, the cumulative incidence for patients were across time above that for controls, except for neoplasms where little differences were found.

Table 2 lists cumulative incidences at selected time points for any somatic disease in the total samples of patients and controls and shows that over 24 years most individuals (93% of the cases and 89% of the controls) had any somatic disease. While there were small differences after two decades, larger cumulative incidences for patients than controls were found closer to entry. For example, at 2 and 10 years the cumulative incidence (95% CI) for patients was .312 (.302–.321) and .723 (.714–.736), while it was .198 (.195–.202) and .623 (.618–.629) for controls.

**TABLE 2:** Cumulative incidences for any somatic disease in the total sample

	Cumulative incidence (95% CI)	
Years after inclusion	Patients	Controls
.5	.132 (.125–.139)	.063 (.060–.065)
1	.207 (.199–.215)	.113 (.110–.115)
2	.312 (.302–.321)	.198 (.195–.202)
4	.470 (.459–.481)	.338 (.333–.342)
6	.574 (.562–.585)	.450 (.445–.455)
8	.658 (.647–.669)	.542 (.537–.547)
10	.723 (.714–.736)	.623 (.618–.629)
12	.782 (.771–.793)	.691 (.686–.696)
14	.820 (.809–.830)	.744 (.738–.749)
16	.852 (.841–.862)	.786 (.780–.791)
18	.877 (.866–.887)	.820 (.814–.825)
20	.899 (.888–.910)	.847 (.841–.853)
22	.910 (.899–.921)	.869 (.863–.875)
24	.926 (.910–.939)	.888 (.880–.895)

For each of the 12 categories of somatic diseases, Table 3 displays adjusted hazard ratios (HRs) for patients compared to controls for the total sample and the three age groups 8–13, 14–17, and 18–32. There were no indications of nonproportional hazards (*p* > .83) for any disease category. About a decade after entry, regardless of age segment, the hazard for any somatic disease among patients was no different to that among controls. For the total sample for example the HR (95% CI) was at 1.03 (.92–1.15) after 11 years. In contrast, the immediate risk among patients was 60% higher the first 2 years after entry (HR = 1.62; 95% CI, 1.55–1.68). Similar patterns were seen for the categories of neoplasms, blood, circulatory system, musculoskeletal, genitourinary, and autoimmune diseases. Persistent differences between patients and controls were found for respiratory, nervous system, and infectious diseases, for which the HR was roughly constant over time, ranging from 1.15 to 1.45. For digestive system and skin and subcutaneous tissue diseases, the HR declined a bit before reaching about a 35% higher hazard among patients after 6 and 3.5 years, respectively.

**TABLE 3:** Hazard ratios (HR) (95% CI) in somatic disease categories (first diagnosis after inclusion) for the comparison of patients to controls in the total sample and in three age subgroups at first diagnosis of anorexia nervosa together with tests for age subgroups interacting with the AN‐status

	All	Age 8–13	Age 14–17	Age 18–32	Interaction
	Patients *n* = 9985, controls *n* = 49,351	Patients *n* = 1724, controls *n* = 8519	Patients *n* = 3809, controls *n* = 18,848	Patients *n* = 4452, controls *n* = 21,984	
	HR^a^ (95% CI)	HR^a^ (95% CI)	HR^a^ (95% CI)	HR^a^ (95% CI)	
Any					
Years after inclusion: 0–2	1.62 (1.55–1.68)	1.69 (1.51–1.88)	1.51 (1.42–1.61)	1.70 (1.61–1.79)	*
Years after inclusion: 3–11	1.18 (1.13–1.24)	1.16 (1.04–1.29)	1.20 (1.12–1.29)	1.19 (1.11–1.28)	*
Years after inclusion: 12+	1.03 (.92–1.15)	1.02 (.79–1.32)	1.03 (.87–1.22)	.99 (.83–1.19)	*
Infectious diseases					
Years after inclusion: 1–3	1.44 (1.29–1.60)	1.67 (1.23–2.26)	1.46 (1.24–1.73)	1.38 (1.19–1.60)	*
Years after inclusion: 4+	1.45 (1.34–1.57)	1.41 (1.16–1.70)	1.34 (1.18–1.53)	1.55 (1.37–1.76)	*
Neoplasms					
Years after inclusion: 0–10	1.47 (1.17–1.85)	1.28 (.61–2.69)	1.43 (.94–2.16)	1.55 (1.15–2.08)	*
Years after inclusion: 11+	1.02 (.78–1.33)	1.73 (.72–4.16)	1.34 (.85–2.13)	.85 (.60–1.20)	*
Blood diseases					
Years after inclusion: 0–10	1.59 (1.38–1.84)	1.24 (.80–1.94)	1.73 (1.37–2.19)	1.59 (1.31–1.95)	*
Years after inclusion: 11+	1.22 (.98–1.52)	1.65 (.95–2.88)	1.03 (.71–1.50)	1.26 (.93–1.70)	*
Endocrine, nutritional, and metabolic diseases					
Months after inclusion: 0–12	2.75 (2.41–3.15)	4.73 (3.31–6.76)	4.51 (3.58–5.69)	1.88 (1.56–2.26)	<.001
Years after inclusion: 1–8	1.04 (.95–1.13)	2.25 (1.79–2.82)	1.31 (1.12–1.52)	.78 (.70–.88)	<.001
Years after inclusion: 9–15	.69 (.62–.77)	.74 (.54–1.01)	.67 (.57–.80)	.70 (.59–.82)	*
Years after inclusion: 16+	.70 (.58–.84)	.45 (.27–.73)	.74 (.57–.97)	.75 (.57–1.00)	*
Nervous system diseases					
Years after inclusion: 0–7	1.30 (1.17–1.43)	1.21 (.92–1.60)	1.42 (1.21–1.66)	1.24 (1.08–1.43)	*
Years after inclusion: 8+	1.25 (1.13–1.39)	1.15 (.85–1.56)	1.35 (1.14–1.61)	1.22 (1.06–1.41)	*
Circulatory system diseases					
Months after inclusion: 0–12	4.01 (3.23–4.99)	10.5 (4.75–23.4)	4.63 (3.21–6.69)	3.26 (2.44–4.35)	*
Years after inclusion: 1–10	1.54 (1.38–1.71)	1.47 (1.06–2.05)	1.70 (1.41–2.05)	1.49 (1.30–1.71)	*
Years after inclusion: 11+	1.22 (1.07–1.39)	1.33 (.90–1.96)	1.25 (.99–1.57)	1.20 (1.01–1.43)	
Respiratory system diseases					
Years after inclusion: 0–2	1.20 (1.08–1.33)	1.41 (1.09–1.82)	.97 (.82–1.14)	1.36 (1.18–1.58)	*
Years after inclusion: 3+	1.14 (1.06–1.22)	.91 (.76–1.09)	1.16 (1.04–1.31)	1.20 (1.08–1.33)	*
Digestive system diseases					
Years after inclusion: 0–5	1.70 (1.59–1.82)	1.80 (1.51–2.16)	1.55 (1.38–1.73)	1.80 (1.63–1.98)	*
Years after inclusion: 6+	1.37 (1.28–1.47)	1.26 (1.06–1.49)	1.38 (1.23–1.54)	1.41 (1.28–1.55)	*
Skin and subcutaneous tissue diseases					
Years after inclusion: <3.5	1.51 (1.35–1.69)	1.85 (1.35–2.52)	1.37 (1.14–1.65)	1.55 (1.33–1.81)	*
Years after inclusion: >3.5	1.34 (1.23–1.45)	1.55 (1.28–1.88)	1.27 (1.11–1.46)	1.31 (1.16–1.48)	*
Musculoskeletal system diseases					
Months after inclusion: 0–12	2.18 (1.99–2.40)	1.54 (1.16–2.05)	1.32 (1.12–1.56)	3.30 (2.91–3.74)	<.001
Years after inclusion: 1–12	1.42 (1.35–1.49)	1.05 (.92–1.20)	1.41 (1.30–1.52)	1.59 (1.48–1.72)	<.001
Years after inclusion: 13+	1.11 (.98–1.24)	1.02 (.74–1.41)	1.01 (.82–1.24)	1.21 (1.04–1.42)	*
Genitourinary system diseases					
Years after inclusion: 0–4	1.43 (1.34–1.52)	1.56 (1.24–1.95)	1.61 (1.44–1.79)	1.34 (1.24–1.46)	*
Years after inclusion: 5–11	1.22 (1.14–1.31)	1.38 (1.15–1.64)	1.07 (.95–1.20)	1.31 (1.19–1.44)	*
Years after inclusion: 12+	1.22 (1.10–1.34)	1.15 (.90–1.46)	1.20 (1.03–1.39)	1.26 (1.09–1.45)	*
Autoimmune diseases					
Years after inclusion: 0–6	1.20 (1.06–1.37)	1.01 (.70–1.46)	1.13 (.90–1.42)	1.30 (1.10–1.53)	*
Years after inclusion: 7+	1.17 (1.01–1.36)	1.46 (.99–2.16)	.89 (.68–1.16)	1.33 (1.08–1.63)	*

Abbreviation: CI, confidence interval.

a Adjusted for period of inclusion, sex, and family income at baseline (in tertiles).

* *p* > .05.

For endocrine, nutritional, and metabolic diseases, the immediate risk for patients was almost three‐fold higher during the first year after entry when compared to controls (HR = 2.75; 95% CI, 2.41–3.15). However, that ratio was reversed to a 30% reduced hazard for patients from 9 years after entry and onwards. Differences across age at diagnosis groups pertained only to endocrine, nutritional and metabolic diseases during the first 12 months until 8 years after entry, with higher HRs for the younger age groups 8–13 and 14–17 group than for age group 18–32. The incidences for these diseases increased in the controls around age 20–25 in the two younger age groups (12 years after entry in the age group 8–13 and 6 years after entry in the age group 14–17). This trend was less clear in the oldest age group due to few cases aged less than 12 at entry. Finally, regarding musculoskeletal system diseases during 12 years after entry, HRs were higher in the age group 18–32 years than in the two younger age groups.

## 4 DISCUSSION

The findings of the present study are based on data from a nationwide cohort of patients with AN and carefully matched controls and on long‐term follow‐up observations. During the first 2 years after entry, there was a 60% higher hazard among the AN patients. From 3 to 11 years the higher risk was reduced to 18%. However, regardless of age at diagnosis, the hazard for any somatic disease became no different among patients compared to controls a decade after AN onset. For all subcategories, the HR for patients compared to controls increased close to entry, with increments ranging from 14% for respiratory system disease to 300% for circulatory system diseases. The HRs all declined to less than 1.50 on longer terms. Similarly, another recent study based on Danish registries with a different design and largely using historical ICD‐8 data found that the relative risks of being diagnosed with a range of medical conditions were higher among those with any type of ED including AN (Momen et al., 2021).

In addition, for most disease categories, age at AN diagnosis did not modify the effect on time to first somatic diagnosis after AN diagnosis. This is a new perspective on the course of AN given the fact that age at onset of AN, particularly during childhood compared to later age, is associated with a higher severity of AN and indicators of poor outcome (Grilo & Udo, 2021). Whereas a more benign clinical outcome of AN per se in adolescence compared to adulthood was observed in older studies (Steinhausen, 2002, 2009; Steinhausen et al., 2003).

Furthermore, among the proportion of AN patients that were less prone to getting a somatic disease of the neoplasm, blood, endocrine, nutritional and metabolic, or musculoskeletal system the first years after AN onset, the hazard of the corresponding disease was akin to that among controls. In contrast, a largely constant HR higher than 1 and less than 1.5 applied to respiratory system, nervous system, and infectious diseases. These findings imply that there are more associations between AN and other somatic disease categories than have been focused on so far in a series of clinical studies (Andersen & Ryan, 2009; Frolich et al., 2017, 2020; Kimmel et al., 2016; Misra et al., 2016; Norris et al., 2016; Romanos et al., 2012; Stheneur et al., 2014; Vestergaard et al., 2002).

The total rate of any somatic comorbidity in patients with AN increasing from 31% during the first 2 years to 93% during the 22 years after entry were even higher than the 25% and 56% rates which we observed in preceding analyses of mental comorbidities in the same sample. However, the HRs were lower for somatic than for mental comorbidities, with some 60% increase for the first 2 years, a small increase during the next 10 years and no increase thereafter for somatic diseases. However, the increase for mental comorbidities was more than 100% at all time points.

In addition to the main somatic disease categories of the ICD‐10 spectrum, the increased risk of comorbidity in patients with AN also pertained to the association between AN and autoimmune diseases. While various hypotheses have been put forward to explain potential etiological associations between these diseases (Acres et al., 2012; Clough, 2011; Fetissov et al., 2002; Raevuori et al., 2014), autoimmune diseases were observed only in a rather small number of patients with AN. This also included the cases with associated DM‐type 1 that were not consistently increased at both follow‐up periods confirming the finding of no significant association of AN with DM‐type 1 (Mannucci et al., 2005). Furthermore, the corresponding cumulative incidence curves of the autoimmune diseases were rather low (about 10% cumulative risk after 20 years) in all three AN age subgroups for both the AN patients and the controls.

Among patients with AN, higher immediate risk of any somatic disease was found during the first decade after AN onset than among controls, a finding that may be explained by the close monitoring of AN patients resulting in a possible surveillance bias. In addition, the consequences of the onset of malnutrition by extremely restrictive eating may have contributed to this finding. However, this is an assumption only since the registries do not contain data on weight or BMI. On the other hand, our findings converge with the observed increased prevalence rates of somatic illnesses in other severe mental disorders (De Hert et al., 2011). It may also be likely that the accumulation of the various diseases represents a heightened general vulnerability for an earlier start of somatic diseases in AN compared to controls. The participants, predominately women, in the control set were found to be at an increased risk of endocrine, nutritional and metabolic disorders from about age 16–18. However, for AN patients a similar pattern was less evident. The relatively low cumulative incidence for controls before the age of 16–18 was reflected in high HR (>4) close to entry and low (about .7) later in the younger age groups. The cumulative incidence was slightly higher for controls after about 16 years after entry. However, for all other categories the cumulative incidence across time for AN patients was always higher or at the same level when compared to controls.

The strengths of the present study include the design with large nationwide study populations including patients, well‐matched controls, and long‐term follow‐up periods. Furthermore, the Danish health system with free access to medical care without individual costs implies low thresholds to receiving medical care and, thus, high representativeness. Most noteworthy, the findings were adjusted for various potential confounders including age, sex, and period of entry.

However, the rather typical denial of the illness in patients with AN may have contributed to rather late admissions to services or even lacking identification of some of these individuals although in the vast majority the clinical symptoms are grave enough for referral to services. As the registers do not include any data regarding the outcome at discharge, unfortunately, there is no information available on recovery from any disease, including AN, so that the relationship of the state of AN in terms of recovery versus endurance or severity cannot be analyzed with sufficient accuracy. Other potential limitations lie in the nature of register studies. All patients were treated in the public health system. There is no obligation to register data on patients treated in the smaller sector of privately practicing physicians. Furthermore, there is no independent verification of the accuracy of diagnoses although several Danish studies have shown that diagnostic validity is high for various disorders (Kessing, 1998; Lauritsen et al., 2010; Löffler et al., 1994; Mohr‐Jensen et al., 2016).

In conclusion, the present study convincingly documents that AN is among the most multifaceted diseases not only represented by mental comorbidities (Steinhausen et al., 2021), but also by somatic multimorbidity requiring extensive medical interventions to avoid life‐courses with disabilities and premature death. Future studies might also address the causal question of whether AN causes people to have an increased burden of somatic diseases, which still lies beyond the potential of the present study.

## AUTHOR CONTRIBUTIONS

**Hans‐Christoph Steinhausen:** Conceptualization; formal analysis; investigation; methodology; project administration; supervision; writing – original draft. **Niels Bilenberg:** Conceptualization; funding acquisition; resources; writing – review and editing. **Martin Dalgaard Villumsen:** Data curation; formal analysis; investigation; methodology; writing – review and editing. **Kirsten Hørder:** Conceptualization; writing – review and editing. **René Klinkby Støving:** Conceptualization; funding acquisition; investigation; project administration; resources; writing – original draft; writing – review and editing. **Laura Al‐Dakhiel Winkler:** Conceptualization; writing – review and editing.

## CONFLICTS OF INTEREST

In the past 3 years, Hans‐Christoph Steinhausen has worked as a speaker for Medice and has received book royalties from Cambridge University Press, Elsevier, Hogrefe, Klett, and Kohlhammer publishers. Other authors declare no conflicts of interest.

## Supporting information


**TABLE S1**Number of somatic diseases for patients with anorexia nervosa and controls during follow‐up

**FIGURE S1**Cumulative incidences with 95% confidence intervals for somatic disease categories of patients with AN and controls by age at inclusion

## Data Availability

Data were provided by Statistics Denmark in an anonymized fashion by request of the authors.
