# Supplementary material for: Increased risk of somatic diseases following anorexia nervosa in a controlled nationwide cohort study
Source: Int J Eat Disord. 2022 Apr 22;55(6):754–62. doi: 10.1002/eat.23718 (PMC9323483; doi:10.1002/eat.23718)
Supplement: Supplementary file 1 — TABLE S1Number of somatic diseases for patients with anorexia nervosa and controls during follow‐up [file EAT-55-754-s001.docx]

## Supplementary Table 1. Number of somatic diseases for patients with anorexia nervosa and controls during follow-up

|  | **All** | | **Age 8–13 at inclusion** | | **Age 14–17 at inclusion** | | **Age 18–32 at inclusion** | |
| --- | --- | --- | --- | --- | --- | --- | --- | --- |
|  | Patients, n=9985 | Controls, n=49351 | Patients,  n=1724 | Controls,  n=8519 | Patients,  n=3809 | Controls,  n=18848 | Patients,  n=4452 | Controls,  n=21984 |
| Any somatic, no. (%) | 6686 (67.0) | 27879 (56.5) | 1026 (59.5) | 4188 (49.2) | 2466 (64.7) | 10426 (55.3) | 3194 (71.7) | 13265 (60.3) |
| Infectious diseases, no. (%) | 1250 (12.5) | 4160 (8.3) | 193 (11.2) | 632 (7.4) | 468 (12.3) | 1650 (8.8) | 589 (13.2) | 1878 (8.5) |
| Neoplasms, no. (%) | 168 (1.7) | 642 (1.3) | 17 (1.0) | 54 (0.6) | 55 (1.4) | 186 (1.0) | 96 (2.2) | 402 (1.8) |
| Blood diseases, no. (%) | 387 (3.9) | 1186 (2.4) | 46 (2.7) | 149 (1.7) | 133 (3.5) | 425 (2.3) | 208 (4.7) | 612 (2.8) |
| Endocrine, nutritional, and metabolic diseases, no. (%) | 1738 (17.4) | 7380 (15.0) | 273 (15.8) | 815 (9.6) | 639 (16.8) | 2530 (13.4) | 826 (18.6) | 4035 (18.4) |
| Nervous system diseases, no. (%) | 1024 (10.3) | 3642 (7.4) | 124 (7.2) | 466 (5.5) | 367 (9.6) | 1248 (6.6) | 533 (12.0) | 1928 (8.8) |
| Circulatory system diseases, no. (%) | 939 (9.4) | 2865 (5.8) | 103 (6.0) | 285 (3.3) | 306 (8.0) | 868 (4.6) | 530 (11.9) | 1712 (7.8) |
| Respiratory system diseases, no. (%) | 1470 (14.7) | 6044 (12.2) | 223 (12.9) | 1004 (11.8) | 528 (13.9) | 2331 (12.4) | 719 (16.2) | 2709 (12.3) |
| Digestive system diseases, no. (%) | 2355 (23.6) | 7609 (15.4) | 340 (19.7) | 1112 (13.1) | 839 (22.0) | 2874 (15.2) | 1176 (26.4) | 3623 (16.5) |
| Skin and subcutaneous tissue diseases, no. (%) | 3064 (30.7) | 10787 (21.9) | 386 (22.4) | 1738 (20.4) | 1078 (28.3) | 4110 (21.8) | 1600 (35.9) | 4939 (22.5) |
| Musculoskeletal system diseases, no. (%) | 1206 (12.1) | 4182 (8.5) | 197 (11.4) | 595 (7.0) | 418 (11.0) | 1570 (8.3) | 591 (13.3) | 2017 (9.2) |
| Genitourinary system diseases, no. (%) | 2897 (29.0) | 11252 (22.8) | 341 (19.8) | 1281 (15.0) | 982 (25.8) | 3919 (20.8) | 1574 (35.4) | 6052 (27.5) |
| Autoimmune diseases, no. (%) | 606 (6.1) | 2184 (4.4) | 81 (4.7) | 277 (3.3) | 181 (4.8) | 749 (4.0) | 344 (7.7) | 1158 (5.3) |
