# Supplementary figures and images for: Increased risk of somatic diseases following anorexia nervosa in a controlled nationwide cohort study
Source: Int J Eat Disord. 2022 Apr 22;55(6):754–62. doi: 10.1002/eat.23718 (PMC9323483; doi:10.1002/eat.23718)

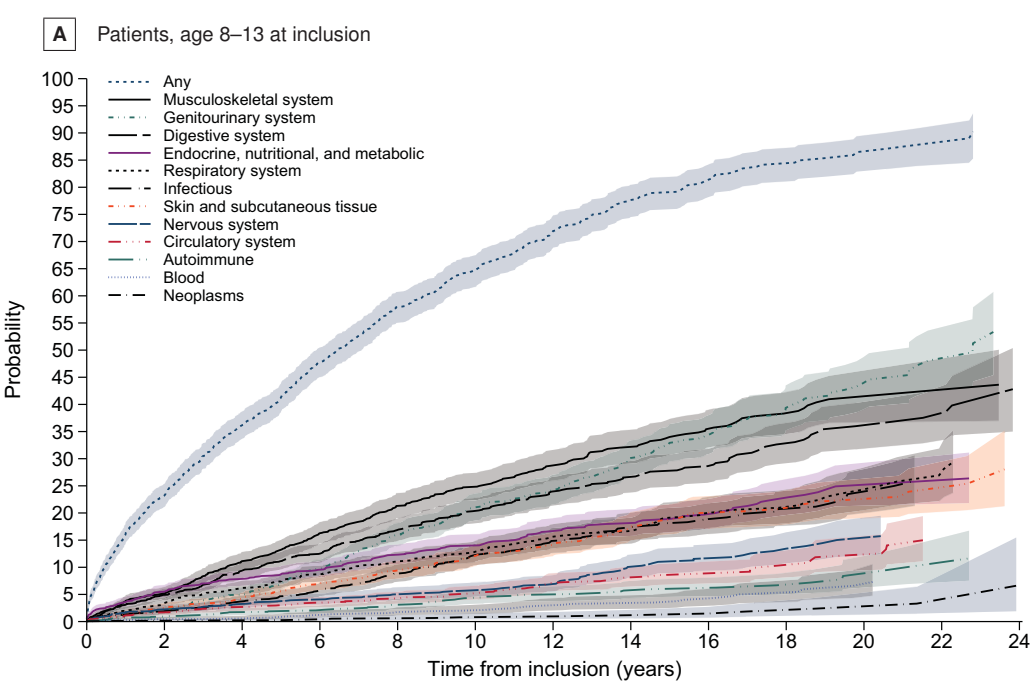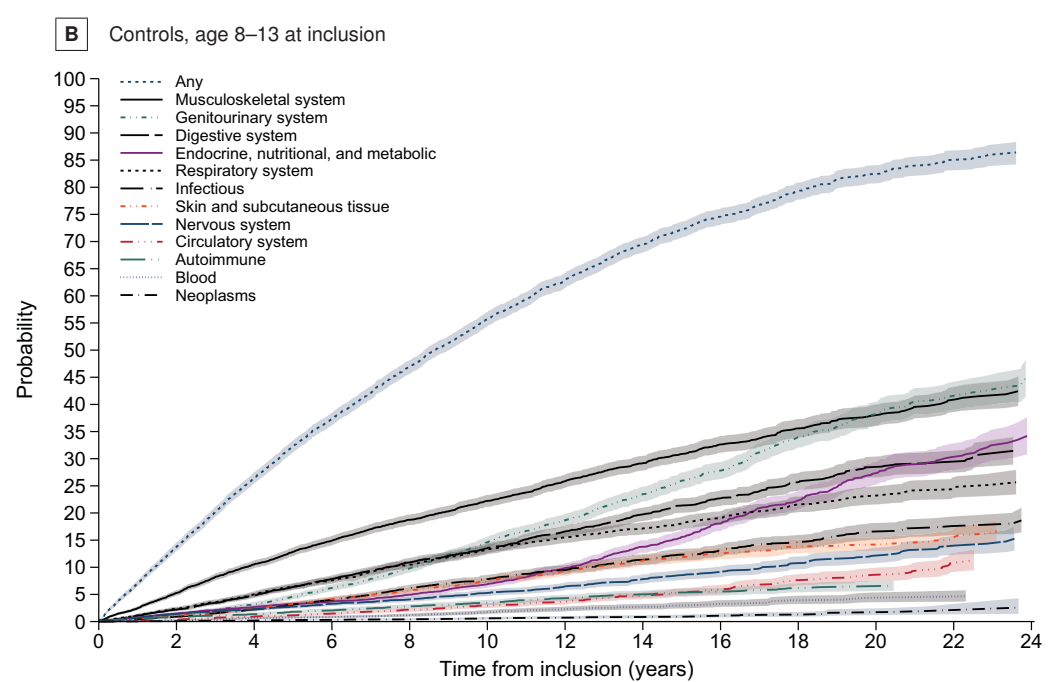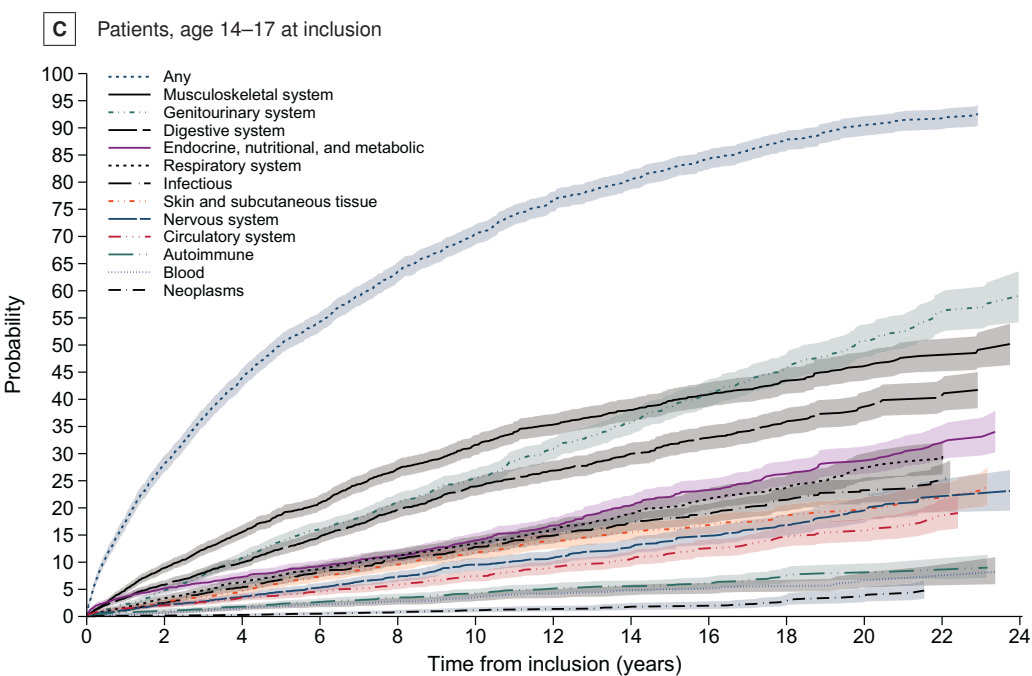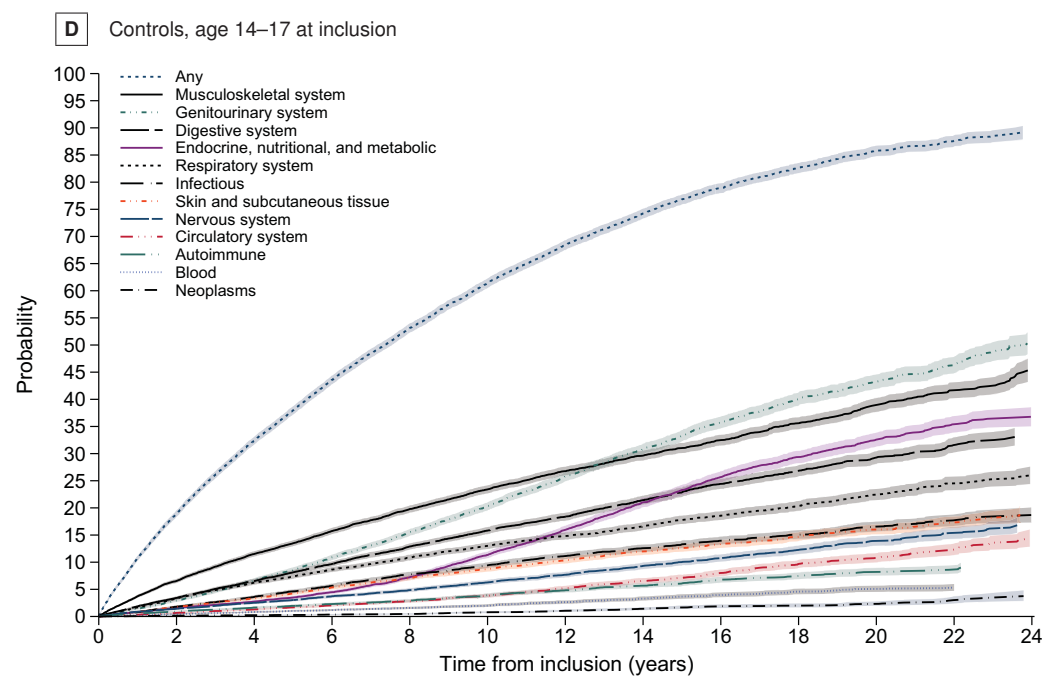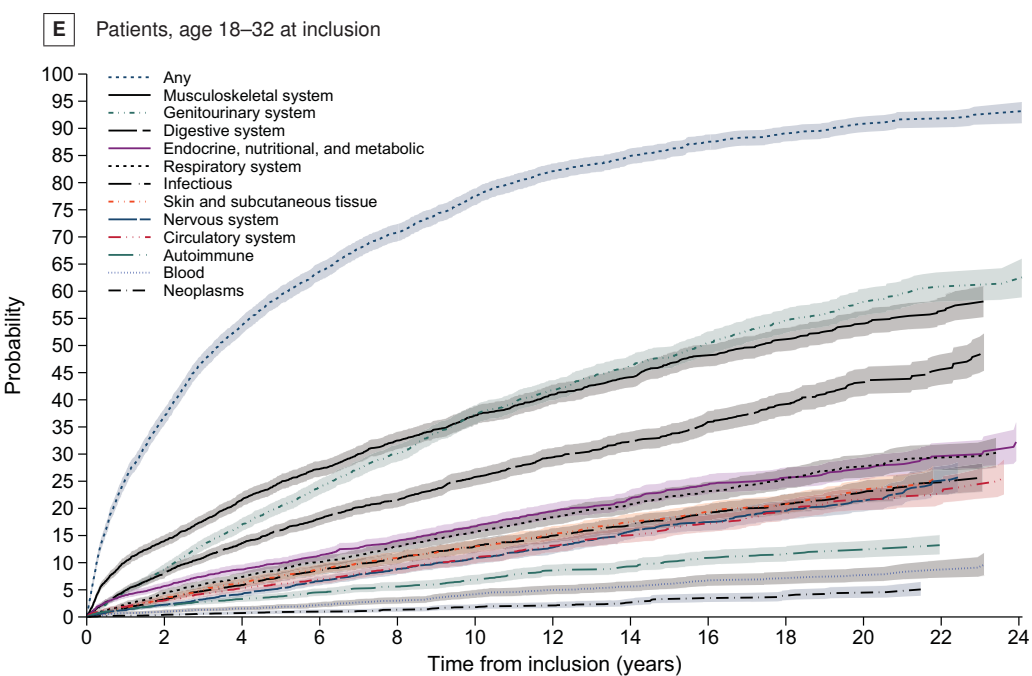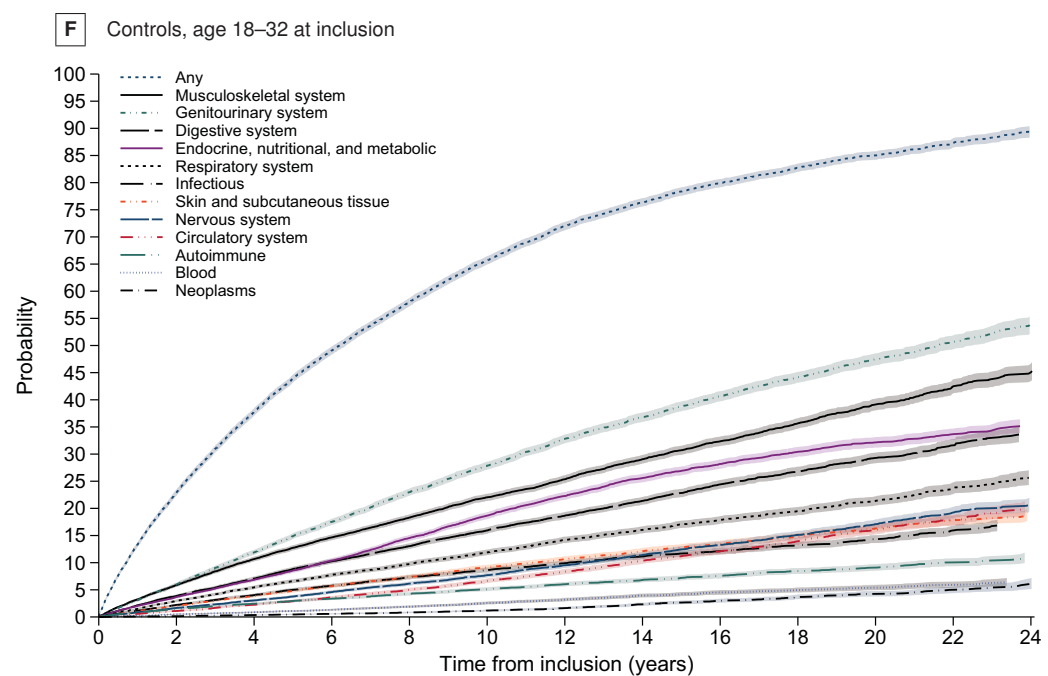

Supplement: Supplementary file 2 — FIGURE S1Cumulative incidences with 95% confidence intervals for somatic disease categories of patients with AN and controls by age at inclusion [file EAT-55-754-s002.pdf]
